# Sphenoethmoidal Meningocele: Endoscopic Approach

**DOI:** 10.7759/cureus.35022

**Published:** 2023-02-15

**Authors:** Naoufal Ramdani, Abdelilah Rguyeg, Drissia Benfdil, Azzedine Lachkar, Fahd Elayoubi

**Affiliations:** 1 Otorhinolaryngology, Centre Hospitalier Universitaire Mohammed V, Faculty of Medicine and Pharmacy, Mohamed First University, Oujda, MAR; 2 Head and Neck Surgery, Centre Hospitalier Universitaire Mohammed V, Faculty of Medicine and Pharmacy, Mohamed First University, Oujda, MAR; 3 Otorhinolaryngology, Head and Neck Surgery, Centre Hospitalier Universitaire Mohammed V, Faculty of Medicine and Pharmacy, Mohamed First University, Oujda, MAR

**Keywords:** meningitis, fat graft, endoscopic surgery, nasosinus scanner, sphenoethmoidal meningocele

## Abstract

The sphenoethmoidal meningocele is a herniation of the meninges through a communication of the skull base with an aeric cavity. It means the presence of an osteomeningeal breach, which is manifested by cerebrospinal rhinorrhea and nasal obstruction. iIs diagnosis is based on a very specific radiological assessment and biology allows the dosage of certain substances to confirm the nature of the cerebrospinal fluid, such as beta-2-transferrin, Once the breach has been found, the endoscopic route exclusively allows the pathology to be treated and the defect to be reconstructed using different materials before the occurrence of serious complications such as meningitis.

## Introduction

Meningocele is defined by a hernia of the meninges through a bone defect of the skull base in a cavity, a rare pathological entity. Its sphenoethmoidal location is exceptional. It can be spontaneous or post-traumatic but poses a problem for management. The endoscopic route endonasal has found its place in the treatment of this pathology.

The particularity of this disease is that it can remain asymptomatic for a long time as it can manifest itself by a serious complication involving the vital prognosis.

## Case presentation

**Figure 1:**
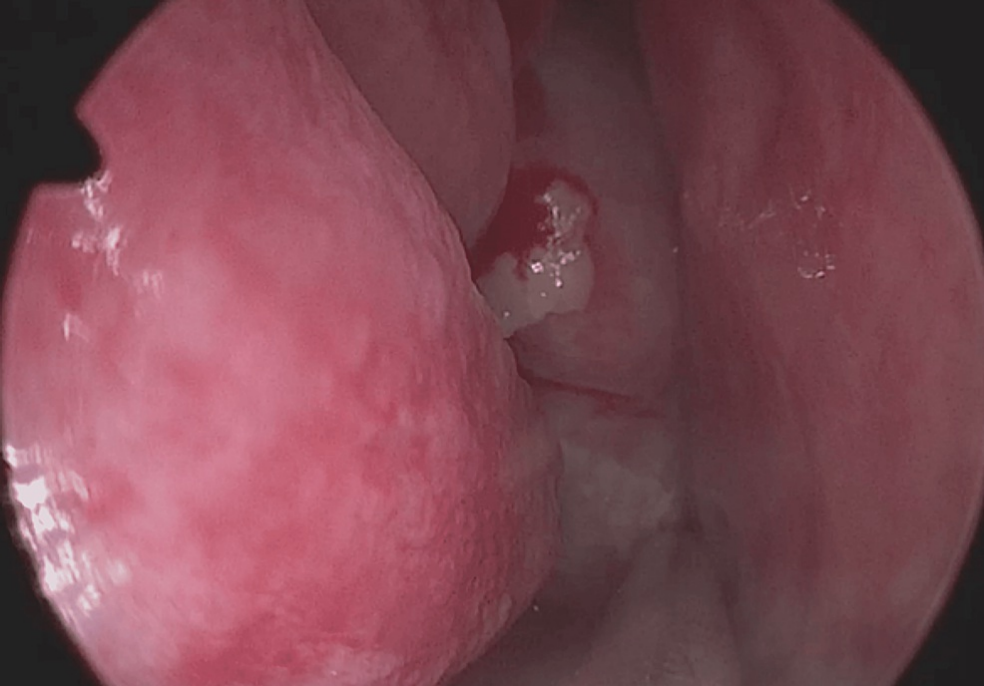
Endoscopic view of the sphenoethmoidal meningocele

**Figure 2:**
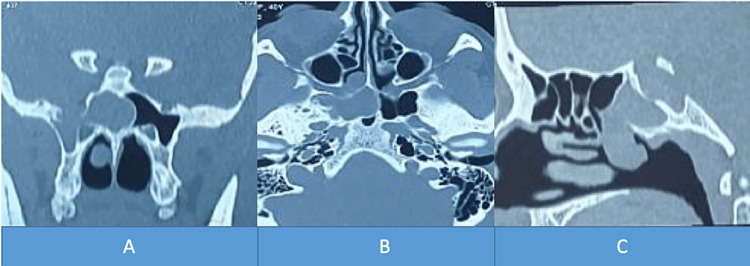
Nasosinus CT sections showing the right sphenoethmoidal meningocele (A: coronal, B: axial, C: sagittal)

**Figure 3:**
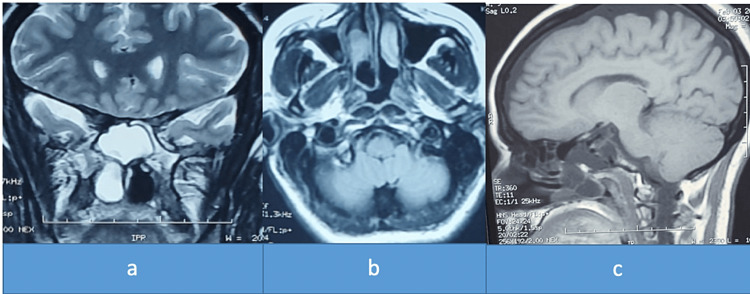
MRI showing the meningocele hyposignal T1 and hypersignal T2 with the same CSF signal in the right nasal cavity

A 42-year-old female, with no significant pathological history, presented with a 5-year-old right chronic nasal obstruction associated with intermittent clear rhinorrhea, complicated 15 days ago of meningitis for which she was treated with antibiotic therapy. The clinical examination found a bluish-pink formation of the posterior-upper part of the right nasal fossa at nasofibroscopy (Figure 1). The rhinorrhea fluid at the test strip was glucose positive and the beta-2-transferrin assay was also positive. The patient benefited from a nasosinusal and cerebral CT that found a homogeneous bridge of the right sphenoidal sinus that descended into the homolateral nasal fossa (Figure 2). MRI showed a hypo-signal T1 and hyper-signal T2 image with the same CSF signal in the right sphenoid sinus that descended through the anterior wall into the homolateral nasal fossa, without herniation of the cerebral parenchyma (Figure 3).

**Figure 4:**
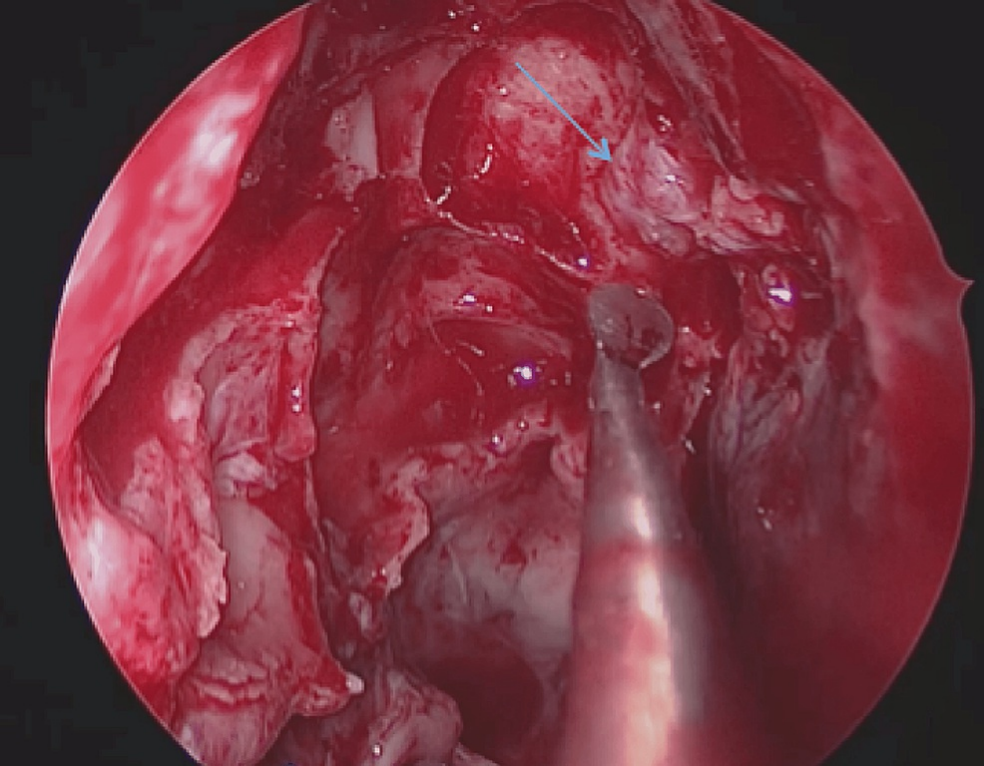
Intraoperative image showing the osteomeningeal breach at the roof of the right posterior ethmoid

**Figure 5:**
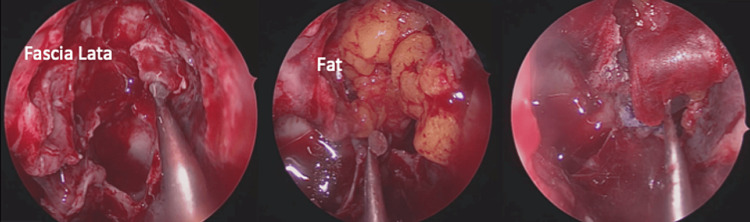
Multilayer closure of the osteomeningeal breach

The patient underwent endoscopic endonasal surgery, which allowed resection of the meningocele and individualization of the osteomeningeal breach (Figure 4) that was reconstructed with abdominal fat, facia lata, and application of biological glue (Figure 5). The surgical intervention lasted two hours and 15 minutes with an estimated blood loss of 300 ml without any intraoperative incident or accident (Video 1).

**Video 1:** Surgical video of the patient with meningocele

The surgical specimen was sent for histological study which confirmed the diagnosis of meningocele. The postoperative course was simple and we performed a lumbar puncture in order to reduce the intracranial pressure, which favored healing and complete closure of the breach. The duration of hospitalization was four days. After 18 months, no signs of recurrence or complications were noted.

## Discussion

There are several forms of meningocele, Van Nouhuys and Bruyn classified them into spheno-ethmoidal, spheno-orbital, trans-ethmoidal or cribriform, sphenomaxillary, and trans-sphenoidal meningocele [1]. Spheno-ethmoidal localization is very rare and it can be spontaneous or secondary to surgery, trauma, or tumor [2]. It can be diagnosed at any age and the clinical manifestations are dominated by nasal obstruction, cerebrospinal rhinorrhea, a complication such as meningitis, or incidentally during imaging done for headache or sinusitis [3]. A paraclinical workup is often necessary to make the diagnosis and to rule out a tumor, beta-2-transferrin assay, and especially beta-trace, allow confirmation that it is indeed cerebrospinal fluid in the rhinorrhea [4]. High-resolution CT has an accuracy of 93-96%, a sensitivity of 92-94%, and a specificity of 100% [5], while MRI can differentiate a meningocele from a meningoencephalocele [6]. In the case of our patient, it was revealed by meningitis and the assessment made by CT scan and MRI showed the meningocele and the bone defect that must be reconstructed.

There are two techniques for the treatment of meningoceles and the reconstruction of osteomeningeal breaches: the classic neurosurgical route and the endoscopic route. The endonasal endoscopic approach has proven to be effective in the treatment of this pathology, highlighting the osteomeningeal breach, resecting the meningocele, and sealing the bony defect [7]. Several materials have been proposed for reconstruction such as abdominal fat, temporal aponevrosis, facia lata, and biological glue; however, most authors prefer fat due to its rapid healing powers as it is more resistant to local infections due to its rapid vascularization and ease of harvesting [8]. In the case of our patient, we noticed a rapid healing with the absence of recurrence. However, the choice between its different materials is based on the seat and the extent of the breach, the proximity of the cisterns to the base of the skull, and the experience of the operating team.

The success rate with the endoscopic approach reaches 80-85% of cases with less morbidity compared to the transcranial approach such as anosmia, frontal retraction and cerebral hemorrhage [9,10].

## Conclusions

Meningocele is a rare pathology that can simulate a nasal tumor and can have serious life-threatening complications. Its diagnosis requires an appropriate workup and the treatment has benefited from the progression of endoscopic endonasal surgery. These minimally invasive procedures have very low morbidity and a success rate close to 90%.

## References

[1] Van Nouhuys JM, Bruyn GW (1964). Nasopharyngeal transsphenoidal encephalocele, craterlike hole in the optic disc and agenesis of the corpus callosum. Pneumoencephalographic visualisation in a case. Psychiatr Neurol Neurochir.

[2] Sakoda K, Ishikawa S, Uozumi T, Hirakawa K, Okazaki H, Harada Y (1979). Sphenoethmoidal meningoencephalocele associated with agenesis of corpus callosum and median cleft lip and palate. Case report. J Neurosurg.

[3] Domengie F, Cottier JP, Lescanne E, Aesch B, Vinikoff-Sonier C, Gallas S, Herbreteau D (2004). Management of cerebrospinal fluid fistulae: physiopathology, imaging and treatment (Article in French). J Neuroradiol.

[4] Wise SK, Schlosser RJ (2007). Evaluation of spontaneous nasal cerebrospinal fluid leaks. Curr Opin Otolaryngol Head Neck Surg.

[5] Shetty PG, Shroff MM, Sahani DV, Kirtane MV (1998). Evaluation of high-resolution CT and MR cisternography in the diagnosis of cerebrospinal fluid fistula. AJNR Am J Neuroradiol.

[6] Reyt E, Righini C, Schmerber S, Karkas A (2011). Cerebrospinal rhinorrhea (Article in French). EMC - Oto-rhino-laryngologie.

[7] Banks CA, Palmer JN, Chiu AG, O'Malley BW Jr, Woodworth BA, Kennedy DW (2009). Endoscopic closure of CSF rhinorrhea: 193 cases over 21 years. Otolaryngol Head Neck Surg.

[8] Trinh VT, Duckworth EA (2015). Scarless abdominal fat graft harvest for neurosurgical procedures: technical note. J Neurol Surg B Skull Base.

[9] Hirsch O (1952). Successful closure of cere brospinal fluid rhinorrhea by endonasal surgery. AMA Arch Otolaryngol.

[10] Zlab MK, Moore GF, Daly DT, Yonkers AJ (1992). Cerebrospinal fluid rhinorrhea: a review of the literature. Ear Nose Throat J.

